# Prognostic Factors Associated With Treatment Failure in Uncomplicated Stercoral Colitis at an Emergency Department: A Retrospective Cohort Study

**DOI:** 10.7759/cureus.84519

**Published:** 2025-05-21

**Authors:** Yuji Okazaki, Toshihisa Ichiba, Yuki Kataoka

**Affiliations:** 1 Department of Emergency Medicine, Hiroshima City Hiroshima Citizens Hospital, Hiroshima, JPN; 2 Department of Systematic Reviews, Scientific Research WorkS Peer Support Group (SRWS-PSG), Osaka, JPN; 3 Department of Internal Medicine, Kyoto Min-iren Asukai Hospital, Kyoto, JPN; 4 Department of Healthcare Epidemiology, Kyoto University Graduate School of Medicine/Public Health, Kyoto, JPN

**Keywords:** computed tomography (ct ), failure treatment, peritonitis, poor prognostic factor, stercoral colitis

## Abstract

Background: Stercoral colitis (SC) can potentially lead to bowel ischemia, sepsis, and perforation. While physicians have increasingly recognized the clinical importance of this condition, the risk of progression in patients with uncomplicated SC who do not have indications for emergency surgery remains unclear. The aim of this study was to determine prognostic factors associated with treatment failure in patients with uncomplicated SC in an emergency care setting.

Methods: We conducted a retrospective cohort study at a tertiary care hospital from April 2013 to March 2023. We included patients aged 18 years or older who were diagnosed with uncomplicated SC based on computed tomography (CT) at the emergency department. We analyzed the following prognostic factors: age, frailty, rebound tenderness, serum amylase level, and D-dimer level. We also analyzed the following CT findings: location of impacted fecaloma, maximum axial diameter and thickening of the affected colon, and the presence of pneumatosis coli and free fluid. The primary outcome was treatment failure, defined as the need for conversion from conservative treatment to emergency surgery or progression to in-hospital death. We used univariate logistic regression analysis to estimate odds ratios (ORs) with 95% confidence intervals (CIs).

Results: Of the 117 patients identified, 15 patients (13%) had treatment failure. The presence of rebound tenderness (crude OR 57, 95% CI 10-330) and CT findings of pneumatosis coli (crude OR 4.7, 95% CI 1.5-14) and free fluid (crude OR 6.5, 95% CI 2.0-21) were associated with treatment failure. Frailty and results of blood examinations were not associated with poor outcomes.

Conclusion: A substantial number of patients with uncomplicated SC progressed to requiring emergency surgery or died. Physical signs of peritonitis and specific CT findings may aid in the identification of high-risk patients who require closer monitoring and potential surgical intervention.

## Introduction

Stercoral colitis (SC) is characterized by focal bowel wall inflammation caused by increased colonic pressure from fecal impaction [1-3]. This condition usually occurs in elderly individuals, patients with neurocognitive disorders, and opioid users [3-5]. Due to the global aging population and the increased use of computed tomography (CT) for examining cases of severe constipation, the number of people diagnosed with SC has been increasing [4]. SC can lead to severe complications such as acute bowel ischemia, sepsis, and bowel perforation, and it has a mortality rate of 17% to 57% [6,7]. Thus, early recognition and appropriate management of SC are needed in emergency care settings. Despite the lack of evidence-based guidance for management, physicians determine whether to choose conservative treatment (e.g., fecal disimpaction and antibiotics) or surgical intervention based on physical examination findings, laboratory results, and CT findings [4,8].

Identification of patients with SC but with no signs of bowel ischemia, sepsis, or perforation (i.e., uncomplicated SC) who truly require surgical intervention is challenging in emergency care settings. For patients with SC, complications such as bowel perforation or the presence of portal venous gas are clear indications for emergency surgery, and elevated lactate levels or high levels of inflammatory markers often lead to surgical intervention [4]. In contrast, distinguishing between ischemia and non-ischemia of the affected colon can be difficult even when contrast-enhanced CT is used [9], and in cases without obvious indications for surgical intervention, physicians are likely to make a diagnosis of uncomplicated SC and choose conservative treatment. However, even in cases that are clinically judged as uncomplicated SC, conservative treatment may fail, leading to the requirement of emergency surgery or death [4]. Despite the clinical importance of risk factors for treatment failure in emergency care settings, there have been few studies in which risk factors for treatment failure, including conversion to emergency surgery following a diagnosis of uncomplicated SC, have been investigated.

To address the knowledge gap, we investigated factors associated with treatment failure for this condition in an emergency care setting. The results may provide insights into risk stratification for patients with uncomplicated SC and guide further appropriate management.

## Materials and methods

Study design and setting

This retrospective cohort study was conducted at a tertiary care hospital in Hiroshima City, Japan, from April 1, 2013, to March 31, 2023. The hospital has 743 beds, and its emergency department receives approximately 7,000 ambulance transports and more than 15,000 walk-in patients annually. The emergency department is staffed by 10 attending physicians. The study was approved by the Institutional Review Board of Hiroshima City Hiroshima Citizens Hospital (approval no: 2023-41). The need for informed consent from participants was waived due to the anonymized nature of the data and the implementation of an opt-out strategy for participant recruitment. To report this study, we adhered to the Strengthening the Reporting of Observational Studies in Epidemiology (STROBE) guidelines (Appendices) [10].

Selection of participants

We focused on patients with uncomplicated SC in this study. We used the following process to select target patients. We selected patients aged 18 years or older who presented to our emergency department (ED) with acute abdominal symptoms and underwent computed tomography (CT) with or without contrast agents and for whom there was a registered disease name based on the following International Classification of Diseases, 10th Revision (ICD-10): A052 (necrotizing enterocolitis), K550 (acute ischemic colitis, subacute ischemic colitis, or fulminant ischemic colitis), K564 (fecal impaction or fecal ileus), and K566 (obstructive ileus or obstructive colitis). Among them, a diagnosis of uncomplicated SC was made based on the following CT criteria: (1) fecal impaction or fecaloma, (2) colonic dilatation, (3) pericolic fat stranding, (4) wall thickening of the affected colon segment, and (5) the absence of indication for emergency surgery including portal venous gas and perforation (Appendices) [11]. The CT diagnosis was based on the radiologist's report and a detailed review of medical records and CT images by an emergency physician (YO) to determine the presence of uncomplicated SC. When a radiologist's report was unavailable, two emergency physicians (YO and TI) independently reviewed the CT images to determine the presence or absence of this condition. One of the reviewers (TI) was blinded to the clinical information and outcomes when interpreting the CT images. If there was disagreement, the two reviewers (YO and TI) discussed the case to resolve the discrepancy. We excluded patients who met the following criteria: (1) obstruction due to solid tumors such as colorectal cancer (i.e., obstructive colitis), (2) only fecal impaction, (3) emergent surgery including exploratory laparotomy, (4) SC with in-hospital onset, (5) terminal-stage cancers, and (6) out-of-hospital cardiac arrest due to SC. If a patient experienced multiple episodes of SC during the observational period, we included only the first occurrence in the study.

Prognostic factors

Based on previous studies and expert consensus, we analyzed the following prognostic factors: age, frailty, rebound tenderness, serum amylase level, and D-dimer level. We also analyzed the following CT findings: location of impacted fecaloma, maximum axial diameter and thickening of the affected colon, and the presence of pneumatosis coli and free fluid [4,5,11,12]. We used all measurements at the time of ED presentation. We assessed frailty by using the Clinical Frailty Scale (CFS), which is a rapid frailty screening tool with scores ranging from 1 (very fit) to 9 (terminally ill) [13]. Two emergency physicians (YO and TI) independently evaluated each CT finding, and in cases of discrepancy, they discussed the findings to reach a consensus.

Outcomes

We assessed treatment failure as the primary outcome. Treatment failure was defined as a composite of change in the initial treatment strategy (i.e., the need for conversion from conservative treatment to emergency surgery or in-hospital death). We included emergency surgery only when it was related to SC as treatment failure. We included any cause of death in our hospital as in-hospital death. We did not include patients who died after transfer to another hospital in in-hospital mortality because the association with SC was unclear, and transfers to hospice care for acute illnesses are rarely performed in Japan. In addition, we assessed in-hospital mortality as the secondary outcome.

Data collection

We obtained the following information for characteristics of the study patients: gender, body mass index, time from symptom onset to ED visit, vital signs (temperature and shock index) [14], comorbidities (heart failure, ischemic stroke, hemorrhagic stroke, dialysis, liver cirrhosis, dementia, and psychiatric disorders such as depression), medications prior to admission (opioids and glucocorticoids), results of laboratory examinations (white blood cell count, C-reactive protein, creatinine kinase, and venous lactic acid), and use of contrast media for CT scanning.

Statistical analyses

We summarized dichotomous variables as percentages, continuous variables as medians and interquartile ranges (IQRs), and categorical variables as means and standard deviations (SDs). Due to the limited information on prognostic factors in uncomplicated SC, we included all available data rather than calculating a formal sample size. We assumed that the data were missing at random and performed multiple imputation. For the imputation process, we incorporated all variables used in the analysis model, including the outcome variable and relevant covariates. We created 100 imputed datasets and analyzed each dataset separately using the analysis model [15]. Subsequently, we applied Rubin’s rule to combine the results from the 100 datasets, yielding the final estimates and their standard errors [16]. To estimate the effect size of potential prognostic factors on the outcomes of interest, we performed univariate analyses. For these analyses, we used a logistic regression model and reported the odds ratios (ORs) with 95% confidence intervals (CIs). To confirm the robustness of the analysis, we performed a sensitivity analysis limited to patients who underwent contrast-enhanced CT. These patients were selected because ischemic changes of the affected colons could be evaluated in detail, leading to an accurate diagnosis of uncomplicated SC. We performed statistical analyses using R software (version 4.3.2; R Foundation for Statistical Computing, Vienna, Austria).

## Results

Characteristics of study participants

**Figure 1:**
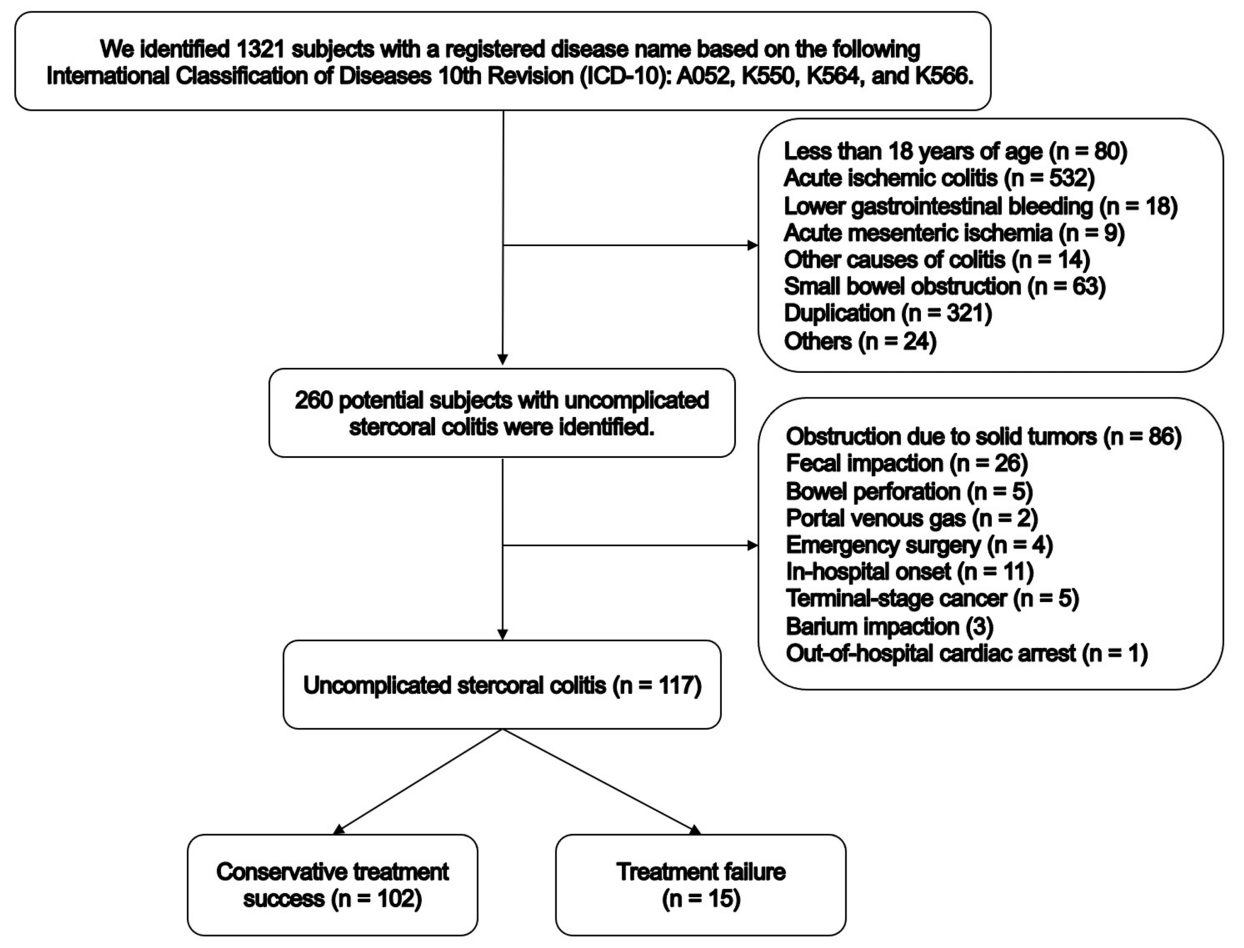
Patient flow chart

We identified 117 patients with uncomplicated SC diagnosed in our ED (Figure 1).

Of those patients, 15 patients (13%) had treatment failure during hospitalization, 9 patients (7.7%) underwent emergency surgery after conservative treatment had been chosen, and 8 patients (6.8%) died in the hospital. Table 1 shows the characteristics of patients in whom conservative treatment was successful and those with treatment failure.

**Table 1:** Characteristics of patients with uncomplicated stercoral colitis The Clinical Frailty Scale is a 9-point scale to assess a person's overall fitness and functional status, with 1 being the best (very fit) and 9 being the worst (terminally ill). Shock index was calculated as heart rate divided by systolic blood pressure. We provided information on missing data only for variables with unmeasured data. IQR: interquartile range, ED: emergency department, SD: standard deviation.

Variables	Conservative treatment success (n = 102)	Treatment failure (n = 15)
Age (years), median (IQR)	77 (68, 86)	83 (75, 90)
Female, n (%)	64 (63)	7 (47)
Body mass index, median (IQR)	20.2 (17.8, 22.9)	20.3 (19.5, 22.4)
Missing data, n (%)	5 (4.9)	1 (6.7)
Symptom onset to ED visit (hours), median (IQR)	4.5 (3.0, 8.0)	3.5 (2.0, 7.0)
Missing data, n (%)	23 (23)	2 (13)
Emergency surgery, n (%)	-	9 (60)
In-hospital death, n (%)	-	8 (53)
Comorbidities		
Heart failure, n (%)	3 (2.9)	1 (6.7)
Ischemic stroke, n (%)	9 (8.8)	4 (27)
Hemorrhagic stroke, n (%)	2 (2)	3 (20)
Dementia, n (%)	18 (18)	2 (13)
Liver cirrhosis, n (%)	1 (1)	0
Dialysis, n (%)	1 (1)	1 (6.7)
Mental disorder, n (%)	15 (15)	1 (6.7)
Medication		
Opioid, n (%)	3 (2.9)	0
Glucocorticoid, n (%)	5 (4.9)	0
Clinical Frailty Scale, mean (SD)	4.2 (2.0)	4.3 (1.8)
Physical values		
Rebound tenderness, n (%)	2 (2.0)	8 (53)
Body temperature (℃), median (IQR)	36.7 (36, 37)	36.6 (36, 37.2)
Missing data, n (%)	-	1 (6.7)
Shock index	0.61 (0.50, 0.78)	0.63 (0.48, 1.15)
Laboratory values		
White cell count (/μL), median (IQR)	13100 (8800, 16300)	14500 (12500, 19600)
Missing data, n (%)	1 (1)	-
C-reactive protein (mg/dl), median (IQR)	0.31 (0.07, 1.2)	1.02 (0.26, 1.34)
Missing data, n (%)	1 (1)	-
Creatinine kinase (U/L), median (IQR)	86 (64, 121)	67 (55, 119)
Missing data, n (%)	10 (9.8)	-
Amylase (U/L), median (IQR)	107 (68, 155)	316 (65, 880)
Missing data, n (%)	22 (22)	5 (33)
D-dimer (μg/mL), median (IQR)	2 (0.80, 4.9)	7.2 (4.3, 34)
Missing data, n (%)	63 (62)	5 (33)
Venous lactate levels (mmol/L), median (IQR)	2.1 (1.5, 3.2)	2.7 (1.8, 4.7)
Missing data, n (%)	9 (8.6)	1 (6.7)

The proportions of patients with ischemic stroke (27%) and hemorrhagic stroke (20%) were higher in the treatment failure group than in the conservative treatment success group. Only 3 patients (2.9%) in the conservative treatment success group were regular opioid users. A comparison of vital signs between the two groups showed that both body temperature and shock index were comparable. The median venous lactate levels measured at ED admission were also not markedly different between the two groups (2.1 mmol/L, IQR 1.5-3.2 vs 2.7 mmol/L, IQR 1.8-4.7). Table 2 shows the CT findings in patients with conservative treatment success and those with treatment failure.

**Table 2:** Computed tomography findings for patients with uncomplicated stercoral colitis: conservative treatment success versus treatment failure *We measured the width and thickness of the affected colon in the axial view of CT. CT: computed tomography, IQR: interquartile range.

Variables	Conservative treatment success (n = 102)	Treatment failure (n = 15)
Contrast-enhanced CT, n (%)	64 (63)	9 (60)
Location of impacted fecaloma		
Rectum, n (%)	56 (55)	4 (27)
Sigmoid colon, n (%)	32 (31)	11 (73)
Descending colon, n (%)	10 (9.8)	0
Transverse colon, n (%)	4 (3.9)	0
Maximum width of the affected colon^* ^(cm), median (IQR)	5.6 (4.3, 6.8)	4.6 (4.0, 6.8)
Maximum thickening of the affected colon wall^*^ (mm), median (IQR)	3.4 (2.6, 4.1)	4.0 (3.2, 4.9)
Pneumatosis coli, n (%)	20 (20)	8 (53)
Free fluid, n (%)	24 (24)	10 (67)

Sixty-four patients (63%) with conservative treatment success underwent contrast-enhanced CT, while 9 patients (60%) with treatment failure underwent the same imaging study.

Associations between prognostic factors and outcomes

Table 3 shows the associations between 10 potential prognostic factors and treatment failure.

**Table 3:** Factors associated with treatment failure in cases of uncomplicated stercoral colitis *Sensitivity analysis was conducted for only subjects diagnosed as having uncomplicated stercoral colitis by contrast-enhanced computed tomography. We conducted univariate logistic regression analyses for each potential prognostic factor. The analysis was also conducted using multiple imputation for missing data. OR: odds ratio, CI: confidence interval.

Variables	Main analysis, crude OR (95% CI)	Sensitivity analysis*, crude OR (95% CI)
Age	1.0 (1.0–1.1)	1.1 (1.0–1.2)
Frailty	0.96 (0.74–1.3)	1.2 (0.82–1.7)
Rebound tenderness	57 (10–330)	39 (5.5–280)
Amylase	1.0 (1.0–1.0)	1.0 (0.99–1.0)
D-dimer	1.0 (0.99–1.0)	1.0 (0.96–1.1)
Location of impacted fecaloma	1.2 (0.6–2.3)	0.99 (0.42–2.3)
Maximum width of the affected colon	0.95 (0.67–1.3)	0.91 (0.42–2.3)
Maximum wall thickening of the affected colon	1.6 (0.96–2.6)	1.8 (0.95–3.5)
Pneumatosis coli	4.7 (1.5–14)	9.6 (2.0–46)
Free fluid	6.5 (2.0–21)	43,000,000 (0 to ∞)

The presence of rebound tenderness was strongly associated with treatment failure (crude OR 57, 95% CI 10-330). Additionally, the presence of pneumatosis coli and free fluid on CT was both positively associated with treatment failure (crude OR 4.7, 95% CI 1.5-14 and 6.5, 95% CI 2.0-21, respectively). Frailty, amylase levels, D-dimer levels, location of the impacted fecaloma, and the maximum width and wall thickening of the affected colon were not associated with treatment failure. The results of the sensitivity analysis confirmed the findings of the main analysis (Table 3).

Table 4 presents the associations between these prognostic factors and in-hospital mortality.

**Table 4:** Factors associated with in-hospital mortality in cases of uncomplicated stercoral colitis *Sensitivity analysis was conducted for only subjects diagnosed as having uncomplicated stercoral colitis by contrast-enhanced computed tomography. We conducted univariate logistic regression analyses for each potential prognostic factor. The analysis was also conducted using multiple imputation for missing data. OR: odds ratio, CI: confidence interval.

Variables	Main analysis, Crude OR (95% CI)	Sensitivity analysis*, Crude OR (95% CI)
Age	1.2 (1.0–1.4)	1.2 (1.0–1.4)
Frailty	1.3 (0.84–1.9)	1.9 (0.89–4.2)
Rebound tenderness	35 (6.3–190)	87 (7.0–1100)
Amylase	1.0 (1.0–1.0)	1.0 (0.99–1.0)
D-dimer	1.0 (1.0–1.1)	1.0 (0.96–1.1)
Location of impacted fecaloma	1.2 (0.5–2.9)	1.0 (0.3–3.1)
Maximum width of the affected colon	1.3 (0.85–2.0)	1.2 (0.70–2.2)
Maximum wall thickening of the affected colon	2.2 (1.1–4.2)	2.6 (1.1–6.2)
Pneumatosis coli	6.2 (1.4–28)	17 (1.7–170)
Free fluid	8.7 (1.6–46)	190,000,000 (0 to ∞)

The presence of rebound tenderness was positively associated with in-hospital mortality (crude OR 35, 95% CI 6.3-190). The presence of pneumatosis coli and the presence of free fluid on CT were positively associated with in-hospital mortality (crude OR 6.2, 95% CI 1.4-28, and 8.7, 95% CI 1.6-46, respectively). Additionally, maximum wall thickening of the affected colon was positively associated with in-hospital mortality (crude OR 2.2, 95% CI 1.1-4.2). Frailty, amylase levels, D-dimer levels, location of impacted fecaloma, and maximum width of the affected colon were not associated with in-hospital mortality. The results of the sensitivity analysis confirmed the main analysis findings for in-hospital mortality (Table 4).

## Discussion

We found that one in eight patients diagnosed with uncomplicated SC in our ED had treatment failure during hospitalization. Physical examination revealing rebound tenderness and CT findings of pneumatosis coli and free fluid were positively associated with both treatment failure and in-hospital mortality. Additionally, increased colonic wall thickening on CT was positively associated with in-hospital mortality. In contrast, frailty and results of blood examinations were not associated with prognosis.

Progression from uncomplicated to complicated SC may occasionally occur. We considered two possible etiologies for treatment failure in this exacerbation: the development of perforation or extensive bowel ischemia [2,17,18]. These complications of SC arise from increased intraluminal pressure caused by fecaloma, which impairs capillary perfusion in the bowel wall, compromising regional vascular supply and transmural perfusion [2,4]. This leads to ulceration and eventual perforation in cases of localized bowel mucosal ischemia [18,19]. In some cases, bowel mucosal ischemia may extensively progress without perforation, leading to sepsis [7,17]. Therefore, even in cases of uncomplicated SC, emergency physicians should recognize that progression of this condition may occur even after conservative treatment such as fecal disimpaction and antimicrobial therapy.

Our study revealed that peritoneal signs on physical examination and CT findings of pneumatosis coli and free fluid were novel prognostic factors in uncomplicated SC. A previous study on nonperforated SC focused primarily on demographic and comorbidity factors, including advanced age, dementia, and low body mass index [12]. While one retrospective study demonstrated an association between increased length of involved colonic segments and mortality [11], our study provides the first evidence that specific physical examination findings and CT markers predict treatment failure in uncomplicated SC. These findings might enable risk stratification for patients presenting with uncomplicated SC, potentially leading to early identification of high-risk cases that require closer monitoring and aggressive intervention.

A proper physical examination might help to predict potential progression to complicated SC. Our study showed that 7.7% of patients with uncomplicated disease required surgical intervention. Therefore, physicians may need a simple, repeatable, and inexpensive prognostic factor such as rebound tenderness. Indeed, the diagnostic accuracy of rebound tenderness in peritonitis may be insufficient, and judgments based solely on physical findings may be unreliable [20]. However, the results of our study demonstrated that vital signs and laboratory findings at the time of the ED visit could not predict poor clinical outcomes. While understanding the limitations of physical findings, the detection of peritoneal signs on physical examination, including rebound tenderness, may serve as one of the most easily available predictors of disease progression. If peritoneal signs are present in emergency care settings or rebound tenderness develops when choosing conservative treatment, emergency physicians should consider consulting with surgeons.

Frailty and blood examinations at ED presentation may not be useful for risk stratification of treatment failure in cases of uncomplicated SC. Frailty assessed by the CFS score has been reported to be a useful risk stratification tool for poor clinical outcomes in elderly patients presenting to the ED [21,22]. The tool is also feasible and easy to use in emergency care settings [23]. However, as our study showed, this may not be applicable to patients with uncomplicated SC. Furthermore, although there was a trend toward elevated levels of amylase and D-dimer, suggestive of intestinal ischemia [24,25], in uncomplicated SC patients who had treatment failure, this study did not identify predictive markers of prognosis. For better risk stratification for the challenging management of this condition, future studies may need to consider the clinical course, including the rate of change in blood examinations, rather than a single measurement at the time of the ED visit.

Another strength of this study is that it revealed that easily identifiable findings on CT (i.e., pneumatosis coli and free fluid) may predict clinical deterioration. Pneumatosis coli on CT is one of the suggestive findings of bowel mucosal ischemia secondary to fecal impaction [4,11]. Furthermore, the presence of free fluid indicates inflammation of the colon leading to peritonitis [4,11]. In other words, these findings may reflect the severity of SC. Ideally, CT images of an acute abdomen caused by a life-threatening condition such as SC should be rapidly transmitted to a radiologist for appropriate interpretation [26]. However, the feasibility of such rapid transmission varies depending on the medical setting and available resources. Therefore, even if a non-radiologist must interpret the CT findings of SC, if physicians can identify one of these readily recognizable findings, they may be able to closely monitor the patient's clinical progression and take timely interventional steps. In addition, based on our sensitivity analysis, our findings without the use of a contrast agent did not affect the main results. Therefore, the results of this study may be equally applicable to patients with an allergy to a contrast agent.

This study has several limitations. First, this was a single-center, retrospective study. Due to the small sample size, we did not conduct multivariate analysis. Additionally, some results showed wide 95% confidence intervals. A study with a larger number of patients with uncomplicated SC is needed to confirm our findings. This limitation may result in underpowered analyses and unstable effect estimates. Second, we were unable to adjust for confounding factors because we did not perform multivariate analysis. Consequently, the identified associations might be overestimated or underestimated. With this in mind, our findings should be interpreted with caution. Third, we diagnosed the target population as having uncomplicated SC based on CT findings rather than pathological findings. Our patients had a higher frequency of rebound tenderness, pneumatosis coli, and free fluid than those in previous studies, which may have influenced the results [8]. We attribute this difference to the fact that all of the patients included in our study had pericolic fat stranding, which may be related to the severity of SC [4,11]. The external validity of these findings should be carefully considered. Fourth, conservative treatment was not standardized in our hospital. The treatment strategy after admission depended on individual physicians. The impact of this limitation on our estimated effect size is uncertain. While there are no international guidelines for managing SC [4], future studies may be needed to predict clinical outcomes based on standardized treatment protocols because they might influence clinical outcomes.

## Conclusions

A substantial number of cases of uncomplicated SC required emergency surgery or resulted in in-hospital death after diagnosis in an emergency care setting. The presence of signs of peritonitis may indicate a potential risk of poor clinical outcome. If such signs are present, careful monitoring of the clinical course is essential. Further studies with a larger sample size and a standardized conservative treatment protocol may be needed to investigate prognostic factors for clinical outcomes in patients with uncomplicated SC.

## Figures and Tables

**Figure 2:**
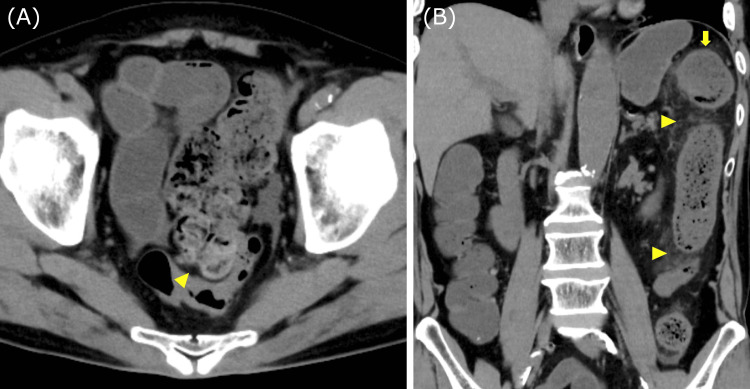
Representative computed tomography findings in a patient with uncomplicated stercoral colitis (A) Computed tomography (CT) image showing fecal impaction in the sigmoid colon (yellow arrowhead). (B) CT image showing pericolic fat stranding (yellow arrowhead) and wall thickening of the dilated segment of the descending colon (yellow arrow).

## Appendices

The checklist of items recommended for cohort study reports is presented in Table 5, and representative computed tomography findings in a patient with uncomplicated stercoral colitis are shown in Figure 2.

**Table 5:** STROBE Statement—Checklist of items that should be included in reports of cohort studies

Category	Item No.	Recommendation	Page
Title and abstract	1	(a) Indicate the study’s design with a commonly used term in the title or the abstract	1
		(b) Provide in the abstract an informative and balanced summary of what was done and what was found	1
Introduction			
Background/rationale	2	Explain the scientific background and rationale for the investigation being reported	1, 2
Objectives	3	State specific objectives, including any prespecified hypotheses	2
Methods			
Study design	4	Present key elements of study design early in the paper	2
Setting	5	Describe the setting, locations, and relevant dates, including periods of recruitment, exposure, follow-up, and data collection	2
Participants	6	(a) Give the eligibility criteria, and the sources and methods of selection of participants. Describe methods of follow-up	2
		(b) For matched studies, give matching criteria and number of exposed and unexposed	Not applicable
Variables	7	Clearly define all outcomes, exposures, predictors, potential confounders, and effect modifiers. Give diagnostic criteria, if applicable	2, 3
Data sources/ measurement	8*	For each variable of interest, give sources of data and details of methods of assessment (measurement). Describe comparability of assessment methods if there is more than one group	2, 3
Bias	9	Describe any efforts to address potential sources of bias	Not applicable
Study size	10	Explain how the study size was arrived at	3
Quantitative variables	11	Explain how quantitative variables were handled in the analyses. If applicable, describe which groupings were chosen and why	
Statistical methods	12	(a) Describe all statistical methods, including those used to control for confounding	3
		(b) Describe any methods used to examine subgroups and interactions	Not applicable
		(c) Explain how missing data were addressed	3
		(d) If applicable, explain how loss to follow-up was addressed	Not applicable
		(e) Describe any sensitivity analyses	3
Results			
Participants	13*	(a) Report numbers of individuals at each stage of study—eg numbers potentially eligible, examined for eligibility, confirmed eligible, included in the study, completing follow-up, and analysed	3
		(b) Give reasons for non-participation at each stage	3
		(c) Consider use of a flow diagram	Figure 1
Descriptive data	14*	(a) Give characteristics of study participants (eg demographic, clinical, social) and information on exposures and potential confounders	3, 5
		(b) Indicate number of participants with missing data for each variable of interest	Table 1
		(c) Summarise follow-up time (eg, average and total amount)	Not applicable
Outcome data	15*	Report numbers of outcome events or summary measures over time	5, 6, 7
Main results	16	(a) Give unadjusted estimates and, if applicable, confounder-adjusted estimates and their precision (eg, 95% confidence interval). Make clear which confounders were adjusted for and why they were included	5, 6, 7
		(b) Report category boundaries when continuous variables were categorized	Not applicable
		(c) If relevant, consider translating estimates of relative risk into absolute risk for a meaningful time period	Not applicable
Other analyses	17	Report other analyses done—eg analyses of subgroups and interactions, and sensitivity analyses	6, 7
Discussion			
Key results	18	Summarise key results with reference to study objectives	7
Limitations	19	Discuss limitations of the study, taking into account sources of potential bias or imprecision. Discuss both direction and magnitude of any potential bias	8
Interpretation	20	Give a cautious overall interpretation of results considering objectives, limitations, multiplicity of analyses, results from similar studies, and other relevant evidence	8
Generalisability	21	Discuss the generalisability (external validity) of the study results	8
Other information			
Funding	22	Give the source of funding and the role of the funders for the present study and, if applicable, for the original study on which the present article is based	10
